# *Ancylostoma ceylanicum* infective third-stage larvae are activated by co-culture with HT-29-MTX intestinal epithelial cells

**DOI:** 10.1186/s13071-017-2513-x

**Published:** 2017-12-15

**Authors:** Caitlin M. Feather, John M. Hawdon, John C. March

**Affiliations:** 1 Department of Biological and Environmental Engineering, Cornell University, Ithaca, NY USA; 2 Department of Microbiology, Immunology and Tropical Medicine, George Washington University, Washington, D.C. USA

**Keywords:** *Ancylostoma ceylanicum*, Hookworm, HT-29-MTX, Intestinal epithelial cells, Activation, iL3, Human, RNA-Seq

## Abstract

**Background:**

Human hookworm larvae arrest development until they enter an appropriate host. This makes it difficult to access the larvae for studying larval development or host-parasite interactions. While there are in vivo and in vitro animal models of human hookworm infection, there is currently no human, in vitro model. While animal models have provided much insight into hookworm biology, there are limitations to how closely this can replicate human infection. Therefore, we have developed a human, in vitro model of the initial phase of hookworm infection using intestinal epithelial cell culture.

**Results:**

Co-culture of the human hookworm *Ancylostoma ceylanicum* with the mucus-secreting, human intestinal epithelial cell line HT-29-MTX resulted in activation of infective third-stage larvae, as measured by resumption of feeding. Larvae were maximally activated by direct contact with fully differentiated HT-29-MTX intestinal epithelial cells. HT-29-MTX cells treated with *A. ceylanicum* larvae showed differential gene expression of several immunity-related genes.

**Conclusions:**

Co-culture with HT-29-MTX can be used to activate *A. ceylanicum* larvae. This provides an opportunity to study the interaction of activated larvae with the human intestinal epithelium.

## Background

Hookworm infection continues to be a problem in developing regions of Asia, Africa and Latin America [1]. Infection with hookworm contributes to the cycle of poverty by causing poor growth, anemia, and impaired school performance in children [1, 2]. Hookworm infection also causes illness in adults, especially in those who are immunocompromised or afflicted by other diseases such as malaria and tuberculosis [3].

There are two major hookworm species of humans: *Ancylostoma duodenale* and *Necator americanus* [4]. *Ancylostoma ceylanicum* is an emerging hookworm of humans in southern Asia [5–7]. Unlike the major hookworm species, *A. ceylanicum* can infect dogs and hamsters. The ability of *A. ceylanicum* to parasitize laboratory animals has made it a useful model for the study of human hookworm infection [8].

Hookworm require their host in order to complete development. Eggs in feces from an infected host hatch in the soil and develop to infective third-stage larvae (iL3). At this point, the larvae arrest and will not resume development until they enter their host. Upon entry to the host, the larvae activate, which entails resumption of feeding, secretion of infection-related proteins, and transcriptional changes [9–13]. Activated larvae migrate to the intestine where they continue development through the fourth larval stage (L4), attach to the intestinal wall, and mature to adulthood. There are two routes by which iL3 larvae can migrate to the intestine [4]. *Ancylostoma* sp., if ingested, can travel through the stomach and directly to the intestine. Alternatively, both *Ancylostoma* and *Necator* sp. can enter through the skin upon contact with contaminated soil and travel through the blood stream to the lungs. From the lungs, they are coughed up, swallowed, and travel through the stomach to the intestine.

Since hookworm only continue development beyond the iL3 stage inside their host, the initiation of parasitism is difficult to study. The signaling events allowing the larvae to transition from free-living iL3 to parasitic L4 are still unknown. Elucidation of these events could allow for the development of novel parasite control interventions and identification of immunomodulatory natural products, as well as further our understanding of hookworm biology [12, 14, 15].

In light of this problem, an in vitro method for the study of activation of *A. ceylanicum* iL3 larvae outside of the host has been developed [12, 16, 17]. In this method, larvae are incubated with canine serum and reduced glutathione, resulting in activation, the earliest stage of larval response to the host environment. Although these worms do activate, they do not continue development to L4. Additionally, recent work has shown that larvae activated by this method do not have the same transcriptional profile as larvae activated in vivo [13, 18].

In order to improve upon the existing in vitro model, we have begun development of an alternative, human model for the activation of iL3. In this model, *A. ceylanicum* iL3 are incubated with human intestinal epithelial cells. Using this model, we have successfully activated iL3 worms, causing them to resume feeding to a level exceeding what is observed after percutaneous infection in vivo [19].

## Methods

### Parasites

An Indian strain of *A. ceylanicum* (USNPC No. 102954) was raised in Syrian hamsters as described previously [20]. Hamsters were maintained at the George Washington University in accordance with institutional animal care and use committee guidelines. Infective *A. ceylanicum* L3 s were recovered from coprocultures after approximately 1 week at 27 °C by modified Baermann technique and stored up to 4 weeks in BU buffer (50 mM Na_2_HPO_4_/22 mM KH_2_PO_4_/70 mM NaCl, pH 6.8) [21] at room temperature.

### Cell culture

HT-29-MTX (from Dr Thécla Lesuffleur, INSERM U560, Lille, France) were maintained in DMEM with 10% fetal bovine serum (FBS) and 1X Antibiotic-Antimycotic (Gibco, Gaithersburg, MD, USA; final concentration of 100 units/ml penicillin, 100 μg/ml streptomycin, and 0.25 μg/ml Amphotericin B) at 37 °C with 5% CO_2_. Prior to seeding trays for *A. ceylanicum* activation assays, cells were treated with 0.25% (*v*/v) trypsin and 0.02% EDTA in phosphate-buffered saline, pH 7.4 (PBS).

### *Ancylostoma ceylanicum* activation assays

HT-29-MTX were seeded into 12-well culture trays (VWR, 10,062-894), with 2 × 10^4^ cells per well. For the first 2 days of culture, the cells were incubated in DMEM with 10% FBS and 1× Antibiotic-Antimycotic (Gibco). On day 3 of culture, serum-free media (DMEM with 1X Antibiotic-Antimycotic, 1× Insulin-Transferrin-Selenium, 1× GlutaMax, and 1× MEM NEAA) was substituted for the previous media. All serum-free media (SFM) supplements were products of Gibco. Cells were maintained in SFM, with media changes every other day. On day 6, day 13, or day 20, *A. ceylanicum* iL3 were prepared and approximately 100 were added to each cell culture well with 2 μl of Fungizone (Gibco). To prepare *A. ceylanicum* iL3 for incubation in cell culture, they were pre-treated with 0.05% sodium hypochlorite in 1× PBS for 3 min and then washed twice in 1× PBS. Where indicated, they were incubated in 1% HCl for 30 min and/or incubated in 5% porcine bile in 1× PBS for 2 h. After each treatment they were washed twice in 1× PBS. The worms were then added to cell culture, buffer, or media and incubated for 36 h at 37 °C and 5% CO_2_. Larvae were incubated for 36 h because previous work has shown that by 36 h the maximum number of larvae are activated [17]. After 36 h, the worms were assayed for resumption of feeding, according to the method described in Hawdon & Schad [16]. Briefly, they were incubated in 2.5 mg/ml FITC-labeled BSA (Invitrogen, Carlsbad, CA, USA) for 2 h at 37 °C and 5% CO_2_, washed in 1× PBS, and observed by fluorescent microscopy. Individuals with a fluorescent intestinal lumen were counted as activated.

For *A. ceylanicum* activation assays in the absence of direct contact with the cells, HT-29-MTX were seeded on the bottom of 6-well transwell insert trays (Costar, 3450), with 2 × 10^4^ cells per well. After day 2, they were maintained in SFM, as described above, for 20 days. On day 6, 13, or 20, pre-treated *A. ceylanicum* iL3 larvae were added to the upper portion of the transwell insert and incubated as described above.

A minimum of three biological replicates were conducted for each data point. Where noted, the percentage of larvae feeding was normalized to the percentage of larvae feeding in the SFM or PBS control, as appropriate for the experiment, with the percentage of larvae feeding in the control group being set as zero prior to averaging the results for each biological replicate. This normalization accommodates for batch-to-batch variability in the activation capacity of the larvae. Two-way analysis of variance was performed to look for interactions between variables. Where no interactions were observed, pairwise comparisons using *t*-tests with pooled standard deviations were performed.

### Treatment of HT-29-MTX with *A. ceylanicum* larvae

HT-29-MTX were seeded and maintained as for the 20-day *A. ceylanicum* activation assays. For treatment of HT-29-MTX cells, *A. ceylanicum* larvae were prepared as above with 0.05% sodium hypochlorite treatment followed by 1% HCl treatment. Buffer washes were performed as described above. On day 20, HT-29-MTX cells were treated with approximately 100 *A. ceylanicum* larvae in 20 μl of buffer or with buffer alone and incubated at 37 °C and 5% CO_2_ for 24 h. After 24 h the cells were harvested for RNA isolation.

### RNA isolation

RNA was isolated from and RNA-Seq performed on a total of four samples. Two samples were HT-29-MTX cells treated with *A. ceylanicum* larvae and two control samples were HT-29-MTX cells treated with buffer alone. Total RNA from the samples was purified using Trizol (Thermo Fisher, Waltham, MA, USA). The purification procedure deviated from the commercial Trizol protocol in the following regards: (i) after the first phase separation, an additional chloroform extraction step of the aqueous layer was performed using Phase-lock Gel tubes (5 prime), (ii) 1 μl of GlycoBlue (Thermo Fisher) was added immediately prior to the isopropanol precipitation, and (iii) the RNA was washed twice with 75% ethanol. RNA sample quality was confirmed by spectrophotometry (NanoDrop) and with a fragment analyzer (AATI Fragment Analyzer). For each sample, 1 μg of total RNA was used for further analysis. PolyA+ RNA was isolated with the NEBNext Poly(A) mRNA Magnetic Isolation Module (NEB).

### RNA sequencing

RNA-Seq was performed using the RNA Sequencing Core at Cornell University. TrueSeq-barcoded RNAseq libraries were generated with the NEBNext Ultra Directional RNA Library Prep Kit (NEB). Each library was quantified with a Qubit 2.0 (dsDNA HS kit; Thermo Fisher) and the size distribution was determined using a fragment analyzer (Advanced Analytical) prior to pooling. Libraries were sequenced on the NEXTseq 500. At least 20 M single-end 75 bp reads were generated per library. Reads were trimmed for low quality and adaptor sequences with Cutadapt v1.8. Reads were mapped to the reference genome UCSC hg19 using Tophat v2.0. Cufflinks v2.2 was used to generate FPKM values and to complete statistical analysis on differential gene expression.

## Results

In designing an in vitro method for the activation of *A. ceylanicum* iL3 larvae, we aimed to mimic the oral route of infection, where iL3 larvae are consumed and pass through the stomach into the small intestine [19]. Therefore, after sterilization in 0.05% sodium hypochlorite, *A. ceylanicum* were treated with 1% HCl to mimic passage through the stomach. After HCl treatment, larvae were treated with 5% porcine bile, in a manner similar to that used by Li et al. [22] in *Trichinella* activation. Larvae would encounter bile acids as they pass through the duodenum.

After these pre-treatments, larvae were washed and incubated with HT-29-MTX intestinal epithelial cells (IECs) for 36 h at 37 °C and 5% CO_2_. HT-29 is a cell line derived from a human colon carcinoma [23]. Colon cancer cell lines, when differentiated, are structurally and functionally similar to the epithelium of the small intestine [24]. Methotrexate-adapted HT-29 cells (HT-29-MTX), when grown to post-confluency, are fully differentiated and composed of mucus-secreting goblet cells and polarized, absorptive enterocytes with brush borders [23–25]. HT-29-MTX cells were cultured in SFM, as previous work has shown that incubation of *A. ceylanicum* iL3 with serum alone is sufficient to induce activation of the larvae [12, 16, 17].

As a measure of activation, we calculated the percentage of the larvae that were feeding after incubation with the IECs. Larvae were counted as feeding if they were observed to have fluorescent protein in their intestines after two-hours of incubation with FITC-BSA.

**Fig. 1:**
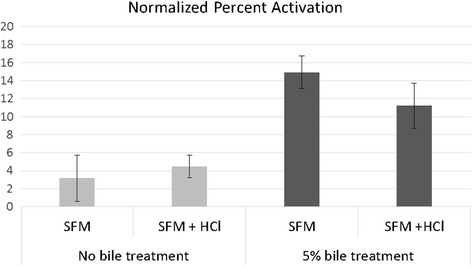
Treatment with 5% bile increases the activation of *Ancylostoma ceylanicum* iL3 larvae. Treatment of *A. ceylanicum* iL3 with 5% bile increases the percent of activated larvae (*P* = 0.0017). Each bar represents the mean of three trials normalized to the percent feeding in PBS alone. Error bars represent the standard error of the mean. Each trial had *n* > 50. Data from each trial was normalized to the percent activation observed in observed in cell-free controls. *Abbreviation*: SFM, serum-free media

First we determined the effect that each step of the treatment protocol had on *A. ceylanicum* activation. Incubation in SFM alone increases the percent of activated *A. ceylanicum* iL3 by 3 %, compared to larvae incubated in PBS. Pre-treatment with HCl did not increase the percentage of feeding larvae over incubation in SFM without pre-treatment (Fig. 1). Although treatment with HCl did not increase the percentage of feeding larvae, we continued to include this step in our larval preparation as an additional sterilization step.

We hypothesized that bile acid pre-treatment may play a role in activation of *A. ceylanicum* iL3. Treatment with bile did significantly increase feeding by approximately 10% (*F*_(1, 9)_ = 16.5379, *P* = 0.0017) (Fig. 1). While bile treatment alone was not sufficient to induce a high level of feeding in the larvae, it may be permissive for activation through other pathways [26]. A two-factor analysis of variance showed no significant interaction between HCl treatment and bile treatment (*F*_(1, 9)_ = 1.2357, *P* > 0.05).

**Fig. 2:**
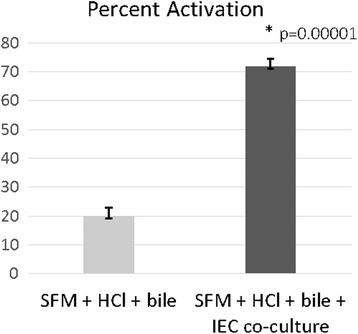
*Ancylostoma ceylanicum* iL3 are activated by co-culture with IECs. Co-culture with HT-29-MTX for 36 h after 1% HCl and 5% porcine bile treatment activates *A. ceylanicum* iL3 (*P* = 0.00001). Error bars represent the standard error of the mean. Each bar represents the mean of three trials, each with *n* > 50. *Abbreviation*: SFM, serum-free media

After treatment with 1% HCl and 5% bile, incubation of *A. ceylanicum* iL3 larvae for 36 h with 21-day cultures of HT-29-MTX results in over 70% of the iL3 becoming activated (Fig. 2). This exceeds the percentage of *A. ceylanicum* iL3 activated by percutaneous infection of hamsters in vivo [19]. Although the larvae did activate, they did not continue development into L4.

**Fig. 3:**
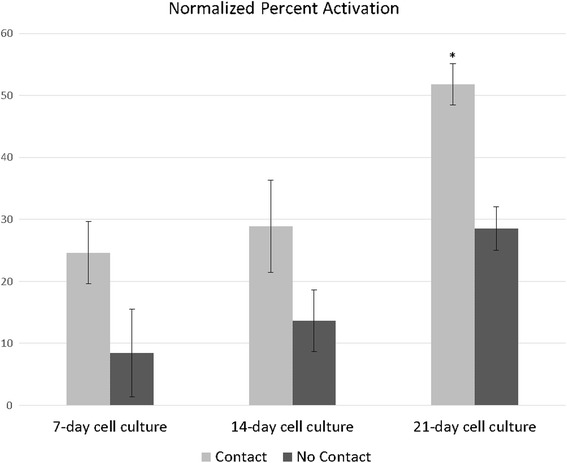
*Ancylostoma ceylanicum* iL3 are maximally activated by differentiated IECs. Mature, 21-day-old cultures of HT-29-MTX maximally activate *Ace* iL3 compared to 7- and 14-day cultures (*P = 0.0058 and *P* = 0.0266, respectively). Direct contact with the cells induces greater activation than no direct contact (*P* = 0.014). Each bar represents the mean of three trials, each with *n* > 50. Error bars represent the standard error of the mean. Data from each trial was normalized to the percent activation observed in observed in cell-free controls

At 21-days, post-confluent cultures of HT-29-MTX are fully differentiated [25]. They express the differentiation-associated proteins dipeptidyl peptidase IV, carcinoembryonic antigen, and villin [23, 24] and have higher expression levels of the mucus protein-coding genes, *muc*s 1-5 [25, 27]. This led us to investigate the effect of age of culture on activation of *A. ceylanicum* iL3 larvae. Twenty one-day cultures of HT-29-MTX are significantly better at activating *A. ceylanicum* iL3 than 7-day or 14-day cultures, *P* = 0.0058 and *P* = 0.0266, respectively (Fig. 3). This difference may be due to the absence or decreased concentration of the activating signal in younger cultures.

The signal from HT-29-MTX that results in activation of may be secreted by the cells into the culture media, or may require direct contact of larvae with the cells. We used transwell inserts to separate larvae from the IECs to test these alternatives. Larvae were activated at a greater percentage with direct contact with the IECs, *P* = 0.014 (Fig. 3). The fact that the larvae were still activated to some extent in the absence of contact suggests that a soluble factor is at least partially responsible for activation of the worms. The reduced percentage of larvae activated without direct contact with the IECs could be due to the soluble factor being at lower concentration in the transwell than next to the cells, or due to some other contact-dependent factor playing a role in activation. A two-factor analysis of variance showed no significant interaction between age of cell culture and direct contact with the culture (*F*_(4, 14)_ = 0.4501, *P* > 0.05).

To investigate the effect that the hookworm have on HT-29-MTX cells, we used RNA-Seq to analyze transcriptional changes in cells treated *A. ceylanicum* larvae. Nineteen genes were identified as differentially expressed, with a *p*-value of 0.00005 and a *q*-value of 0.03. These genes are listed in Table 1.

**Table 1:** Gene expression changes in IECs treated with A. ceylanicum iL3. Using RNA-Seq, gene expression was analyzed in HT-29-MTX cells treated with *A. ceylanicum* larvae and compared to controls (buffer treatment)

NCBI entrez gene ID	NCBI entrez gene ID	HT-29-MTX FPKM	HT-29-MTX + *A. ceylanicum* FPKM	Fold change	Reference
DEFB1 (defensin beta 1)	1672	9	3	-1.5	[41]
SUCNR1 (succinate receptor 1)	56670	4	2	-1.1	[42]
IFI44 (interferon-induced protein 44)	10561	2	4	1.0	[43]
LOC100190986 (uncharacterized)	100190986	3	7	1.0	
PLK2 (polo-like kinase 2)	10769	46	24	-0.9	[44]
NEAT1 (nuclear paraspeckle assembly transcript 1)	283131	115	210	0.9	[45]
ARL14 (ADP ribosylation factor like GTPase 14)	80117	22	12	-0.9	
PFKFB3 (6-phosphofructo-2-kinase/fuctose-2,6-biphosphatase 3)	5209	16	28	0.8	[46]
APCDD1L (APC down-regulated 1 like)	154284	5	3	-0.8	
ADRA2A (adrenoceptor alpha 2A)	150	4	3	-0.7	
ADM (adrenomedullin)	133	71	116	0.7	[47]
DUSP6 (dual specificity phosphatase 6)	1848	13	8	-0.7	[48]
ATF3 (activating transcription factor 3)	467	19	29	0.7	[49]
LFNG (LFNG O-fucosylpeptide 3-beta-N-acetylglucosaminyltransferase)	16858	37	24	-0.6	[50]
HES6 (hes family bHLH transcription factor 6)	55502	37	24	-0.6	
EPHB3 (Eph receptor B3)	2049	8	5	-0.6	[51]
DDX60 (DexD/H-box helicase 60)	55601	0	1	1.9	[52]
NLRC5 (NLR family CARD domain containing 5)	84166	1	1	0.9	[53]
TACSTD2 (tumor-associated calcium signal transducer 2)	4070	35	52	0.6	[54]

*Notes*: For each condition, *n* = 2. Genes were identified as differentially expressed that had a *p*-value equal to 0.00005 and a q-value equal to 0.03. These genes are listed in above. For each gene, a citation that documents the gene’s role in immunity was listed, where applicable

## Discussion

Identification of the host-specific factor responsible for promoting development of hookworm from iL3 to L4 has remained elusive. Similarities between the life cycle of *Caenorhabditis elegans*, a well-studied, free-living soil nematode which can enter an arrested stage of development called ‘dauer’ , and that of hookworm, which constitutively arrest development until entry into the host, has led researchers to look for parallels in the signaling mechanisms between dauer exit and the iL3-L4 transition [28].

A bile acid-like, steroid hormone, dafachronic acid (DA), is required for dauer exit in *C. elegans* through its binding of the nuclear receptor, DAF-12 [26]. *Ancylostoma ceylanicum* lack a homologue of the cytochrome P450 enzyme, DAF-9, which is required for synthesis of DA, although they do have a DAF-12 homologue. Therefore, it has been speculated that a host-derived enzyme may play the role of DAF-9 for synthesis of the DAF-12 ligand [28]. Alternatively, a host-derived bile acid-like molecule could be the DAF-12 ligand. *C. elegans* DAF-12 ligands are capable of inducing feeding in iL3, although not development to L4 [28]. Non-endogenous ligands have been shown to be weak activators of *C. elegans* DAF-12 [29] and the two *C. elegans* DAF-12 ligands are required at higher concentration to activate parasite DAF-12 [28]. This suggests that in hookworm an alternative endogenous or host-derived ligand may better activate DAF-12 and result in development to L4. As the site in the host where the hookworm resume development is the small intestine, we hypothesized that a host bile-acid may be the ligand. To test this hypothesis, we tested if porcine bile acids would have any effect on activation of *A. ceylanicum*, as dog, hamster, and human bile acid are not commercially available. We found that treatment with porcine bile acid only marginally increased *A. ceylanicum* feeding. Since bile acids from a non-host species can induce some level of *A. ceylanicum* feeding, it is possible that host bile acids would be more effective and could contain a DAF-12 ligand.

Co-culture with HT-29-MTX IECs was effective in activating *A. ceylanicum* iL3 larvae, although it did not lead to development to L4. HT-29-MTX do express genes from the cytochrome P450 superfamily, however, they do no express all of the P450 cytochrome genes that are expressed in the human ileum or liver [30]. Therefore we cannot reject the hypothesis that a host P450 enzyme is responsible for the development of iL3 to L4.

As an alternative to the hypothesis of a conserved dauer exit/iL3-L4 signal, the signal for triggering development of iL3 to L4 could come from other cell types present in the intestine. The intestinal epithelium also contains enteroendocrine cells, Paneth cells, stem cells, M cells, and is home to all of the cell types found in the gut-associated lymphoid tissue. Any of these could produce the signal for the iL3-L4 transition. Preliminary work with intestinal organoids derived from human pluripotent stem cells showed that incubation of iL3 larvae with the organoids did not promote development to L4 (data not shown), although further work could be done to optimize conditions.

*Ancylostoma* larvae can infect via a cutaneous or oral route. When infecting via the oral route, larvae do not activate prior to development through L4 [19]. Only when larvae infect via the cutaneous route do they activate and resume feeding prior to arrival in the intestine. It is thought that when larvae infect percutaneously they need to resume feeding to provide energy for their journey to the intestine [19]. In our assay, we aimed to replicate the oral route of infection; however, larvae resumed feeding but did not develop to L4. This suggests that signal for development to L4 is missing from the HT-29-MTX culture and that the larvae perceive the *intestinal* epithelium provided in our model more generally as epithelium.

Despite this, the model we have described here can be used to further study activation of iL3. Larvae activated in the presence of an epithelium may more closely resemble larvae activated in vivo than those activated by serum and reduced glutathione alone. This model provides an opportunity to study the signaling events occurring in the hookworm and the epithelium as they interact. We have taken a first step in this direction by analyzing the transcriptional changes that occur in HT-29-MTX cells treated with *A. ceylanicum* larvae. Of the 19 differentially expressed genes that we identified, 14 have been previously shown to play some role in immunity, including in parasitic infection, bacterial infection, and epithelial barrier function, suggesting that our in vitro system is representative of at least some in vivo events of infection. References for these previous studies are listed in Table 1.

Ht-29 has been used in other immunological studies, both of innate and adaptive immunity [31–37]. There has been some evidence that HT-29 cells respond differently to certain stimuli than do differentiated short-term primary cultures and intestinal epithelial cells in vivo [36, 37] and that HT-29 cells exposed to inflammatory mediators and cytokines produced by cells of the adaptive immune system show behavioral changes [31, 38–40]. Use of an immortalized cell line and lack of feedback from cells of the adaptive immune system may account for why we did not observe large changes in gene expression of HT-29 cells upon exposure to activated hookworm larvae.

## Conclusions

This work is the first to show that human IECs are capable of inducing feeding of iL3 human hookworm larvae. Larvae are maximally activated by direct contact with fully-differentiated HT-29-MTX cells. Incubation with HT-29-MTX cells may provide a better model of iL3 activation than the current model, since the larvae are exposed to a more complex signaling environment than they encounter during activation by serum and reduced glutathione alone. Additionally, this model provides the opportunity to study the interactions between the activated parasite and the host tissue.

## Abbreviations

DA: Dafachronic acid
HT-29-MTX: Methotrexate-adapted HT-29 cells
IECs: Intestinal epithelial cells
iL3: Infective third-stage larvae
L4: Fourth-stage larvae
PBS: Phosphate-buffered saline
SFM: Serum-free media
